# Preferences and expectations of end-users from a mental health educational portal: A qualitative study

**DOI:** 10.34172/hpp.43077

**Published:** 2024-10-31

**Authors:** Sara Pourrazavi, Somayeh Azimi, Ali Fakhari, Habibeh Barzegar, Mostafa Farahbakhsh

**Affiliations:** ^1^Research Center of Psychiatry and Behavioral Science, Tabriz University of Medical Sciences, Tabriz, Iran; ^2^Department of Health Education & Promotion, Tabriz University of Medical Sciences, Tabriz, Iran

**Keywords:** Mental health, Patient preference, Portals, Qualitative research

## Abstract

**Background:**

Digital technologies play an essential role in health systems by providing new solutions to reduce the burden of mental illnesses and disorders. However, in many cases, user preferences and expectations are not considered in the design of portals. This study aims to explore the preferences and expectations of end-users from the features and capabilities of mental health educational portals.

**Methods:**

This qualitative study was conducted from January 2022 to January 2023 using the conventional content analysis approach. The participants were 20 individuals, selected through purposive sampling, ranging in age from 18 to 61, all of whom had prior experience using an educational portal. Data were collected via individual semi-structured interviews. Qualitative analysis was performed using MAXQDA 10 software.

**Results:**

After analyzing the data, the preferences and expectations of end-users were categorized into five main themes: to be reliable, mutual interaction capability, to be accessible, creating a stylish and attractive design, and attention to the quality and structure of the content.

**Conclusion:**

Considering the expectations and needs of users will enhance their acceptance and satisfaction with the portals. From the end-users’ perspectives, the content, appearance, and structural or technical features a mental health educational portal are crucial for its effectiveness.

## Introduction

 Mental disorders are one of the leading causes of global health burden, a situation exacerbated by the COVID-19 pandemic and has contributed to the current global mental health crisis.^1,2^ Although, according to the World Health Organization estimates, nearly one billion people worldwide suffer from mental disorders^3^ and addressing mental health needs is recognized as a human right,^1^ relatively few people in the world have access to appropriate and high-quality mental health services.^3^ On the other hand, provision of mental health services is often disrupted, as was the case during the COVID-19 pandemic. In addition, despite the growing need, many individuals do not seek or utilize mental health services for various reasons, including stigma and discrimination.^3,4^ These factors place significant strain on the healthcare system.^4^ Therefore, health systems require reorientation to address these ongoing challenges.

 In this regard, digital technologies have the potential to assist health systems by offering new solutions to reduce the burden of mental illnesses.^4^ Literature highlights the advantages of digital technologies in improving health services and systems.^5-7^ Digital technologies can simplify and enhance access to mental health services, while also increasing the personalization and flexibility of these services.^4^ Among these technologies, web-based health portals have proven effective in improving patient adherence to treatment, increasing autonomy and participation in treatment, enhancing efficiency of mental healthcare system, reducing medical errors, and improving communication between patients and healthcare providers.^8,9^

 Portals can be applications or websites that provide remote health services, such as offering information, online consultations, and access to electronic patient records.^8,9^ Despite the benefits of portals and the interest patients have in using them, several challenges and barriers have been identified. For instance, patients are concerned about privacy violations by healthcare providers,^9-11^ and healthcare providers worry that patients’ access to their clinical notes may lead to misunderstandings or disagreements with medical decisions.^12,13^ Additionally, many barriers to portal adoption and use have been reported. These include a lack of internet skills, discomfort with computers, preference for in-person communication, mistrust in digital messages, complexity of the language, lack of access to relevant information, and unawareness of the existence and benefits of portals.^9,14^

 In many cases, user preferences and expectations are not adequately considered when designing web-based educational portals. If a portal is not user-centric, users may abandon it, potentially leading to costly redesign. Therefore, it is essential to consider the experiences and needs of various end-users during the design process. The purpose of this study was to explore the preferences and expectations of end-users regarding the functionalities and features of an web-based educational portal for mental health.

## Methods

###  Study design and participants

 This qualitative study explored the expectations and preferences of end users regarding a web-based mental health educational portal from January 2022 to January 2023 in Tabriz, Iran. A total of 20 participants (12 females and 8 males), aged 18–61, who were users of web-based educational portals, took part in individual semi-structured interviews. Participants were recruited using the purposive maximum variation sampling method. The inclusion criteria were first-hand experiences related to the study objectives, prior use of educational portals, and the ability to articulate their experiences and perspectives. The study followed the Consolidated Criteria for Reporting Qualitative Research (COREQ) guidelines.^15^

###  Data collection and analysis

 The interviews were conducted in the respondents’ homes or workplaces, either in person or by phone, and were digitally recorded and transcribed. Each interview lasted roughly one hour and followed established guidelines in informal manner. First, participants were asked to explain, in their own words, what they expected from a mental health education portal. The main questions were: “In your opinion, what features and capabilities should a mental health educational portal have?” and “What makes a good mental educational portal?” Further explanations were also obtained based on participants’ responses and by asking probing questions such as “Would you please explain more about your expression”? The time and location of the interviews were determined by mutual agreement between participants and interviewers. The interviews were continued until data saturation was obtained meaning no new concepts emerged.^16^

 Data analysis was performed concurrently with data collection using conventional qualitative content analysis. Interviews were transcribed verbatim professionally. All interview transcripts were uploaded into the MAXQDA 10 software for coding. The first author developed the initial codebook of themes, which was reviewed and updated by other members of the research team as additional themes emerged.

###  Rigor

 The four criteria of credibility, confirmability, dependability, and transferability suggested by Lincoln and Guba in 1985 were used to evaluate the rigor of the study.^17^ Credibility was ensured through the researcher’s prolonged engagement with the project and consistent interaction with participants. In addition, findings were shared with some participants for feedback to enhance credibility. To ensure confirmability, parts of the interview transcriptions, along with the extracted codes and categories, were reviewed by three external individuals familiar with the qualitative research methods. Dependability was ensured by conducting interviews promptly, accurately recording all stages, and providing equal conditions for all participants. To enhance the transferability, a diverse group of participants was selected based on age, gender, marital status, education, and employment status.

## Results

 After conducting 16 interviews, no new themes were identified, leading us to conclude that the saturation point has been reached. However, we continued conducting interviews, bringing the total to 20 participants. The participants had a mean age of 37.4 years, ranging from 21 to 64. Table 1 presents the demographic characteristics of the participants.

**Table 1:** Demographic characteristics of the participants

**Participant**	**Age**	**Gender**	**Education**	**Occupation**
P1	27	Female	Postgraduate	Midwife
P2	42	Female	Bachelor’s	Nurse
P3	35	Female	Ph.D.	Faculty member
P4	44	Female	Postgraduate	Nurse
P5	48	Female	Ph.D.	Health vice-chancellor employee
P6	32	Female	Bachelor’s	Housewife
P7	21	Male	Bachelor’s	Student
P8	62	Male	Associate diploma	Retired
P9	46	Male	Diploma	Self-employment
P10	27	Male	Bachelor’s	Self-employment
P11	33	Male	Diploma	Self-employment
P12	57	Female	Bachelor’s	Housewife
P13	36	Female	Bachelor’s	Housewife
P14	64	Male	Diploma	Retired
P15	60	Female	High school diploma	Housewife
P16	42	Female	Ph.D.	Psychologist
P17	20	Male	Bachelor’s	Student
P18	43	Female	Diploma	Self-employment
P19	30	Male	Associate diploma	Self-employment
P20	22	Male	Bachelor’s	Unemployment

 We identified 5 themes and 16 sub-themes representing user expectations from mental health education portals. The main categories included are to be reliable, mutual interaction capability, to be accessible, creating a stylish and attractive design, and attention to the quality and structure of the content (Table 2).

**Table 2:** Summarizing the main results

**Main categories**	**Sub-categories**
To be reliable	Ensuring honesty and transparency
	Providing evidence-based information
	Importance of user privacy protection
Mutual interaction capability	To be accountable
	Forums for open discourse
To be accessible	Fast and easy to navigate
	Easy and convenient to access
Creating a stylish and attractive design	Having basic and simple structural architecture
	Having an attractive visual guide
	Personalization
Attention to the quality and structure of the content	Diversity of contents
	Classification of contents
	High quality of the contents

###  To be reliable

 Participants expressed concerns about the authenticity of online content. They believed that a web-based portal for mental health education should prioritize reliability. They identified key reliability criteria, including ensuring honesty and transparency, providing evidence-based information, and protecting user privacy.

####  Ensuring honesty and transparency

 Transparency about individuals involved in the portal’s design and clear articulation of the portal’s objectives and vision were considered essential prerequisites for developing a reliable mental health educational platform. Offering concise and accurate information would instill users confidence in the expertise and reliability of the source. Participants emphasized the importance of users being informed about the designers of the portal through the details available on the portal itself. Providing comprehensive information about the expertise and professional background of the designers and supervisors responsible for the content would enhance the acceptance of published content. As one participant stated:

*“… Having a section listing the names of the responsible individuals is preferable for a portal. For example, it should mention who oversees the material, updates the portal, and the designers. Everything should be transparent.” *(Participant No. 1)

####  Providing evidence-based information

 Providing evidence-based information was another extracted subcategory. Participants felt confident in the authenticity of an educational portal if it presented evidence-based information, cited its sources extensively, and had a solid reputation in the field. In this regard, one of the participants mentioned:

*“…The portal’s material needs to be evidence-based, and its sources should be noted so that users can verify the article’s scientific credibility and consult the cited sources if they need more information.” *(Participant No. 3).

 Another participant highlighted the portal’s reputation:

*“… If the portal is well-known, I am more likely to believe what it says.” *(Participant No. 5).

####  Importance of user privacy protection

 Several interviewees emphasized that the mental health education portal should respect users’ privacy, especially since they may be dealing with mental health issues, and keep their information confidential. One interviewee stated:

*“…No one likes it when their private information gets out, especially considering the social stigma that surrounds mental illness in our culture. Only doctors who have taken an oath to keep their patients’ information private should have access to my medical records through that portal.” *(Participant No. 16)

###  Mutual interaction capability

 Many participants believe that interactivity is crucial to the success of an educational portal dedicated to mental health. The portal should be designed to enable interaction between its creators, mental health professionals, and the users. Ongoing responses from experts to users’ mental health concerns or assistance from portal creators and administrators are examples of this communication. Conversations in discussion forums provide another way portal users can communicate and interact.

####  To be accountable

 Users usually have a lot of inquiries about the educational portals and their contents. Therefore, having personnel available or developing strategies to address their concerns and facilitate better navigation is essential.

####  Having user guide section

 Participants emphasized the importance of an expanded support and guidance section, such as a frequently asked questions section, so that users can find answers to their inquiries independently:

*“…The portal can feature a section labeled “frequently asked questions” or something similar to provide users with support. If users have a similar query, they may search the site and find the solution.” *(Participant No. 3)

####  Existence of specialized consulting section

 Users of the mental health education portal seek answers regarding their mental health. They believe that direct communication with mental health professionals such as psychiatrists and psychologists, is a critical component of a successful mental health portal. One participant emphasized the benefits of virtual consultations via portal:

*“…It is essential that portal have a built-in online assistance system. For example, someone under significant psychological stress should be able to talk to someone and receive guidance immediately.” *(Participant No. 1).

####  Survey and answering questions

 One way to identify areas of a portal that need improvement, and how to enhance it from the users’ perspective, is by conducting polls and providing answers to users’ questions about the content and features of the portal:

*“… I also believe it’s essential to include a section for readers’ feedback at the end of article. People with mental illnesses, in particular, may offer unique insights into the site’s content or functionality. A user poll section would be helpful. The portal’s creators should review and incorporate feedback where it makes sense.” *(Participant No. 6)

####  Forums for open discourse

 The ability for users to interact with each other and share the experiences, especially among those with similar mental issues, has been recognized as a valuable feature of the portal, provided that an expert moderates the conversations. One participant mentioned:

*“…Forums for open discourse, moderated by an expert, are preferable. For instance, if a doctor successfully treats a patient with panic attacks, that method could be useful for other as well.” *(Participant No. 11)

###  To be accessible

 The mental health educational portal should be easily accessible, allowing users to find the content they need quickly. Users highlighted two subcategories: fast and easy to navigate and easy and convenient to access.

####  Fast and easy to navigate

 Portal users should have a seamless experience in quickly locating essential information without having to search extensively within the portal. This ease of use will ensure user satisfaction. Regarding this issue, one user expressed:

*“... A person with a mental disorder and frail nerves must be able to access the data without navigating through multiple pages. The process should take him a few clicks with the mouse to get the required data. Quick page loads are expected. Users of health portals tend to be impatient, searching the portal for immediate answers to their medical questions. If it takes them longer than, say, five to seven seconds to find what they need, they will look elsewhere.” *(Participant No. 3)

####  Easy and convenient to access

 An educational portal should be convenient and straightforward to use. Several participants shared their views on this:

***“…*** *It should not be necessary to register to access the portal’s content, as a user experiencing high mental stress will not have the patience to sign up. Some individuals may also have concerns about Privacy or even paranoia, so they won’t want their information accessible to the general public.* ***”*** (Participant No. 1)

###  Creating a stylish and attractive design

 Participants emphasized that one of the most important factors in attracting visitors to the portal is its design. They recommended that the mental health education portal be designed with a strong technological foundation, incorporating a stylish appearance customizable settings to engage users.

####  Having Basic and simple structural architecture

 The user-friendliness of a portal depends on its layout. If a portal lacks guiding principles or is overly complex, it can confuse users and even cause them to abandon it. A well-designed structure makes it easy for users to access and navigate the portal. One participant stressed the importance of simplicity by saying:

*“…Some portals have such complicated structures that even experienced users struggle. A portal’s layout should not be confusing. For example, it should clearly indicate what users will find on it.” *(Participant No. 5)

####  Having an attractive visual guide

 When creating a mental health education portal, aesthetics and visual guides are equally crucial. Participants raised various details such as the pages’ color and scheme, typeface, font size, texture, and background:

*“…Texts need to be engaging and concise. Utilize a variety of fonts in both small and large sizes. Bright, warm colors tend to attract people more. Different pages, for instance, should have distinct color schemes. Portal users, especially those facing mental health challenges, may feel less mental strain when reading difficult topics if they are presented in bright, varied colors.” *(Participant No. 4)

####  Personalization

 When creating a mental health education portal, it is essential to consider that different users have different needs. The portal’s customizable settings should allow users to securely store personal and medical information and tailor the portal to their individual preferences. One participant highlighted this by saying:

*“…Users should be able to create profiles on the portal, providing personal details such as age, gender, and phone number, so we can content them if their suicide risk assessments indicate a need. They can upload test results, giving advisors instant access to their full medical history. This profile can track medical history including prescriptions, doctors, and hospitalizations.” *(Participant No. 3)

###  Attention to the quality and structure of the content

 The educational portal’s content must be organized into distinct categories to be as helpful as possible. Participants emphasized the importance of high-quality, varied content presented in written, audio, and video formats covering a wide range of themes.

####  Diversity of contents

 Using content that covers diverse topics in written, audio and video formats can enhance user engagement and learning. Interviews between Patients and doctors, as well as with patients’ family members, can support disease management and therapy, as noted by some participants:

*“…Interviews, such as those between psychologists and their patients, have significant value. Videos, audio recordings, and animations can be available online for people to learn from and enjoy. This indirectly guides and advises users.” *(Participant No. 5)

####  Classification of contents

 Participants suggested the deferent categories for organizing contents of mental health education portal. They proposed categorizing the content based on the user’s search intent, different stages of life, and alphabetically. One participant said:

*“…The content must be segmented for various audiences. Is the user looking for Autism symptoms? Looking for a treatment? Additionally, an individual may wish to improve their health. This must be broken down. Prevention, treatment, and follow-up should each be handled separately.” *(Participant No. 4)

####  High quality of the contents

 Participants also emphasized the importance of high-quality material. They expect the portal’s content to maintain an academic tone, be concise, and be regularly updated. One participant stated:

*“…The content should neither be so colloquial and literary that it loses its scientific value, nor so technical and specialized that individuals without the relevant expertise cannot understand it.” *(Participant No. 3)

## Discussion

 Our study explored the preferences and expectations of the end users from an educational portal focused on mental health. Considering the widespread use of health information technology today, it is crucial to tailor this technology to meet user requirements.^18^ Patient portal experiences have demonstrated that failure to align the portal with user expectations can lead to its failure.^19^ Participants discussed the content, visual design, and structural elements they considered essential for a mental health education portal.

 According to our findings, one of the most critical features of the educational portal for mental health is its reliability. Participants identified honesty and transparency, evidence-based information, and user privacy protection as key criteria for ensuring a portal’s reliability. As a primary source of information, the Internet provides easy access to health information today. People expect to find reliable and trustworthy information online. However, the rapid and unsupervised growth of health information on the Internet often raises concerns about its accuracy, leading to user mistrust.^20^ The medical community has expressed concerns over the reliability of online resources, citing the dangers of relying on inaccurate information from unverified sources.^21^ Distrust is often cited as a reason for the low use of online portals.^21^ It is recommended that trust and its effects be carefully considered when designing health websites and portals.^22^ For example, Sarkar et al found that patients often do not trust health applications and are dissatisfied with their design and navigation. They suggested that incorporating feedback from real users into the development process would be beneficial.^23^

 Another theme that participants expected the mental health education portal to provide is capability for mutual interaction. The Internet allows people to consult online with doctors and other healthcare professionals. Patients can use information portals to enhance their interactions with healthcare providers.^24^ Online counselling enables patients to ask questions,^25^ particularly those they may feel embarrassed to ask in person or forgot to ask during office visits.^26,27^ Online consultation also allows doctors to diagnose conditions and recommend necessary therapies^25^ while improving their care suggestions based on the patients’ background information.^28^ Furthermore, web-based platforms enable users to communicate with one another, sharing their health-related experiences.^29^ These features increase user satisfaction with information portals^24,28^ and can enhance engagement with these web-based platforms.

 One of the most crucial characteristics of a mental health educational portal, and one of the most important criteria for the quality of web-based health portals, is how easily users can access the portal and its content.^30^ The participants in this study frequently highlighted this criterion. Users of portals often complain about the complicated registration processes,^31^ and they believe that accessing and using the portal should be simple and straightforward.^32^ However, this is not always the case. Barbara et al acknowledge that while portal accessibility is a strength, users may struggle with tasks like registering or navigating the portal.^33^ Complexity or difficulty to navigate have been shown to reduce users’ understanding of the content, increase confusion, and ultimately lead to dissatisfaction.^32^

 Users also care about the portal’s layout and how it appears on their browsers. Studies have shown that consumers may be dissatisfied with and reject electronic health systems if they are poorly designed.^34^ Baldwin et al assert that creative and user-friendly design can increase the number of people using and accepting portals. Some users may lack health literacy and may evaluate e-health information based solely on appearance of the electronic resource that provide it. As a result, essential health information can be overlooked if the design is poor.^18^

 Participants believed that all diagnostic and treatment options, including their benefits, drawbacks, and potential side effects, should be clearly and comprehensively presented to patients in accordance with the patient’s bill of rights. Educational portal users should be provided with information about the disease, its prognosis, complications, how to contact a doctor, and any training necessary to continue treatment, following this bill of rights.^35,36^ The simplicity and readability of content are just as important as the variety of information offered. The material on educational portals must comply with the Plain Writing Act of 2010, ensuring that structure, paragraph length, and style meet readability standards.^32^

## Limitations

 Our findings should be considered in light of several methodological limitations. The data were translated from Turkish-Azeri to Persian in data transcription and then from Persian to English. As a result, there is a possibility some meaning was lost in translation. Additionally, only individuals who signed informed consent participated in the study, therefore, the sample might be biased toward those people who were willing to share their perspectives. Since it was carried out in a single city in Iran with a specific local context, the ability to apply the findings to broader populations is limited.

## Conclusion

 Our research identified various content, aesthetic, and structural or technical aspects that end users considered essential for a mental health educational portal. Participants emphasized the need for reliability, mutual interaction capabilities, accessibility, a stylish and attractive design, and attention to the quality and structure of the content. Given the large number of individuals using health information technologies such as web-based portals, it is crucial to design portals that meet users’ needs. End-users, particularly those with mental health issues, should be prioritized in the design process, as this will enhance the portal’s acceptance, satisfaction, and successful use.

## Implications for future research

 As health promotion educational portals must be designed to meet the expectations of end users to gain their trust, our approach can serve as a user-centered framework for their development and evaluation. Our future research will implement and evaluate these design principles and assess their impact on users’ health information seeking intentions. Additionally, by presenting the features of a user-centered mental health educational portal, our study can assist healthcare providers in recommending suitable portals to their clients.

## Acknowledments

 We would like to thank the Clinical Research Development Unit of Zahra Mardani Azari Children Educational and Treatment Center, Tabriz university of medical sciences, Tabriz, Iran, for their assistance in this research.

## Competing Interests

 The authors declare no conflict of interest.

## Ethical Approval

 The ethics committee of Tabriz University of Medical Sciences approved the study protocol (Approval ID: IR.TBZMED.REC.1402.049). All participants provided written informed consent before the interviews. Moreover, verbal informed consent was obtained before audio recording. Prior to conducting the interviews, participants were informed about the study’s objectives and methods, and their privacy and anonymity were assured.
